# Geographic Variation and Environmental Predictors of Nontuberculous Mycobacteria in Laboratory Surveillance, Virginia, USA, 2021–2023^1^

**DOI:** 10.3201/eid3003.231162

**Published:** 2024-03

**Authors:** Brendan Mullen, Eric R. Houpt, Josh Colston, Lea Becker, Sharon Johnson, Laura Young, Jasie Hearn, Joe Falkinham, Scott K. Heysell

**Affiliations:** University of Virginia, Charlottesville, Virginia, USA (B. Mullen, E.R. Houpt, J. Colston, L. Becker, S. Johnson, S.K. Heysell);; Virginia Department of Health, Richmond, Virginia, USA (L. Young, J. Hearn);; Virginia Polytechnic Institute and State University, Blacksburg, Virginia, USA (J. Falkinham III)

**Keywords:** nontuberculous mycobacteria, Mycobacterium abscessus, Mycobacterium avium complex, respiratory infections, tuberculosis and other mycobacteria, epidemiology, water vapor, Virginia, United States

## Abstract

Because epidemiologic and environmental risk factors for nontuberculous mycobacteria (NTM) have been reported only infrequently, little information exists about those factors. The state of Virginia, USA, requires certain ecologic features to be included in reports to the Virginia Department of Health, presenting a unique opportunity to study those variables. We analyzed laboratory reports of *Mycobacterium avium* complex (MAC) and *M. abscessus* infections in Virginia during 2021–2023. MAC/*M. abscessus* was isolated from 6.19/100,000 persons, and 2.37/100,000 persons had MAC/*M. abscessus* lung disease. *M. abscessus* accounted for 17.4% and MAC for 82.6% of cases. Saturated vapor pressure was associated with MAC/*M. abscessus* prevalence (prevalence ratio 1.414, 95% CI 1.011–1.980; p = 0.043). Self-supplied water use was a protective factor (incidence rate ratio 0.304, 95% CI 0.098–0.950; p = 0.041). Our findings suggest that a better understanding of geographic clustering and environmental water exposures could help develop future targeted prevention and control efforts.

Nontuberculous mycobacteria (NTM) infections are increasing globally and have thus become pathogens of substantial public health concern (*1*). However, because of scarce public health reporting, little is known about epidemiologic and environmental risk factors for NTM. Virginia is one of the few states in the United States where NTM infections are reported to a statewide public health agency (*2*); those data are uniquely suited to study the NTM bacterial complex. In addition, Virginia, which has areas of varying population density and a relatively large population using self-supplied domestic water (e.g., well water, rainwater captured in cisterns), presents a particularly advantageous location to study the environmental epidemiology of NTM, given its location in the southeastern United States, a region previously described as having a relatively high burden of NTM disease and that has areas of various geographic and climatic conditions: the Coastal Plains (Tidewater), Piedmont, Blue Ridge Mountains, Valley and Ridge, and Appalachian Plateau regions (*3*,*4*).

Exposure to environmental and in-home water sources, soil conditions and metallic content, climate, and coexisting medical conditions are thought to play complex roles in the acquisition and development of NTM infection (*5*). Numerous risk factors for NTM disease have been identified, including coexisting conditions such as compromised immunity, cystic fibrosis, prior cavitary lung disease, and bronchiectasis; atmospheric water vapor content has also been identified as a predictor of NTM rates across cystic fibrosis centers (*6*,*7*). 

Previous studies of NTM epidemiology, often relying on data from retrospective review of electronic medical record databases, suggest NTM are increasing in incidence; the most common pathogens of clinical respiratory disease belong to *Mycobacterium avium* complex (MAC) and *Mycobacterium abscessus* (*8*–*10*). To date, information for epidemiologic research from laboratory surveillance for NTM such as MAC and *M. abscessus* has not been accessed as frequently as for some other pathogens of public health concern (*11*–*15*). Despite this, population-based studies of NTM have found that 86% of patients meeting the American Thoracic Society/Infectious Diseases Society of America microbiologic definition of NTM lung disease also met full clinical criteria for that disease, suggesting microbiologic laboratory-based data could be used for public health surveillance (*16*). We aimed to characterize the geographic distribution of MAC/*M. abscessus* isolates that met microbiologic criteria for NTM lung disease across Virginia to determine geographic clustering and model population-level determinants of prevalence at the county level. For this epidemiologic study, we used demographic and microbiologic data from routine electronic laboratory reports made to the Virginia Department of Health during June 2021–March 2023, as part of a prospective surveillance study approved by human subject review boards at the University of Virginia (#HSR 200234) and Virginia Department of Health. 

## Methods 

The time period for our study encompassed multiple years of inherent seasonality inclusive of all months for which complete data were available from the state health department. These reports included any culture positive for MAC or *M. abscessus* from any laboratory within the state of Virginia. For all positive cultures, we obtained the person’s age, sex, and residential ZIP (postal) code, as well as the anatomic site of sample isolation and date of test result. Case counts were aggregated to the county level based on residential postal codes.

To investigate potential climatic and geographic factors associated with MAC/*M. abscessus* prevalence, we obtained mean annual saturated vapor pressure, mean daily maximum temperature, and mean annual precipitation data for each county in Virginia during 2021–2022 from Weather Source (https://weathersource.com). We extracted the percentage of each county using self-supplied groundwater from US Geological Survey data from 2018, the most recent data available (*4*). Based on a recent US Geological Survey analysis, water source data from Virginia has been reliably recorded and relatively stable over time (*17*). 

### Case Definitions

We defined cases of MAC/*M. abscessus* lung disease using 2020 American Thoracic Society/Infectious Diseases Society of America microbiologic criteria for NTM pulmonary disease (*18*). Case-patients had either a single MAC or *M. abscessus* culture isolated from bronchoalveolar lavage, pleural fluid, or lung tissue or ≥2 cultures from sputum. For persons with multiple cultures collected over time, we included case data only from the earliest culture meeting these criteria. We excluded data from mixed MAC and *M. abscessus* cultures or from successive cultures testing positive for one then the other. We excluded cases not meeting the microbiologic criteria for lung disease in which only 1 sputum culture contained MAC or *M. abscessus*. We excluded data from lung disease cases diagnosed based on nonrespiratory samples. We also excluded data from persons residing outside of Virginia. 

### Statistical Analyses

We analyzed differences in age of MAC and *M. abscessus* case-patients using Mann-Whitney U tests and differences in sex using χ^2^ tests. We obtained US Census Bureau data on population size, median age, and population density for each Virginia county from 2022, the midpoint of the study period (*19*). We calculated average annual prevalence of MAC/*M. abscessus* lung disease captured by laboratory surveillance during 2021–2023 for the entire state of Virginia and for each county and independent city. Average annual prevalence was reported as rate per 100,000 population. 

We generated choropleth maps to visualize total county-level MAC/*M. abscessus*, MAC, and *M. abscessus* infections, saturated vapor pressure, and percentage of county population using self-supplied water. Self-supplied water comes from nonpublic groundwater or surface water sources, such as wells or rainwater captured in cisterns. To assess clustering, we calculated Moran *I* for each map as a measure of spatial autocorrelation. We analyzed factors potentially associated with prevalence of MAC/*M. abscessus* infections in each county using negative binomial regression, a generalization of Poisson regression, to account for overdispersion. We adjusted population numbers using the natural log of person-years as an offset variable. We defined person-years as the given population (e.g., statewide, county) multiplied by 3 years (i.e., length of the study period). We included additional variables in the final model as potentially relevant epidemiologic confounders and environmental factors noted in previous investigations of NTM: sex, median age, population density, mean saturated vapor pressure, mean maximum temperature, mean daily precipitation, and percentage of population using self-supplied water (*3**,**6**,**8**,**10*). We reported exponentiated coefficients from the model as prevalence ratios. We analyzed data using SPSS Statistics 28.0 (IBM, https://www.ibm.com) and generated maps using ArcGIS 3.0 (Environmental Systems Research Institute, https://www.esri.com). 

## Results 

### Statewide Results

We identified 874 persons with >1 MAC or *M. abscessus* pulmonary cultures during the 2021–2023 data collection period. We excluded 10 persons who resided outside of Virginia, leaving data from 864 persons to evaluate. We categorized 714 persons (82.6%) with MAC and 150 (17.4%) with *M. abscessus*; 331/864 (38.3%) of those met microbiologic criteria for NTM lung disease. 

### Case Demographics 

**Figure 1:**
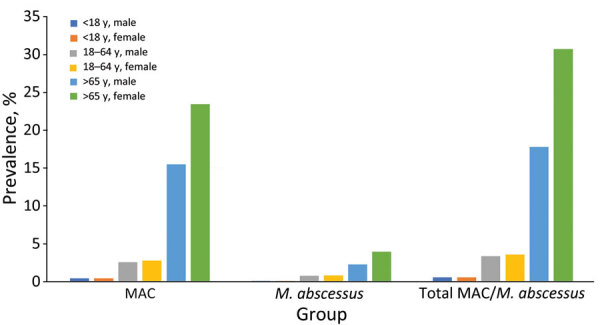
Prevalence of *Mycobacterium avium* complex (MAC), *M. abscessus*, or both (MAC/*M. abscessus*), categorized by age and sex, Virginia, USA, 2021–2023.

Median age was 69 (interquartile range [IQR] 58–76) years among case-patients identified with MAC/*M. abscessus* infections overall, median 64 (IQR 46–75) years among those with *M. abscessus*, and median 69 (IQR 60–77) years among those with MAC. Only 18 case-patients (2.1%) were <18 years of age, and 534 (61.8%) were >65 years of age. Sex distribution for all case-patients was 497 (57.5%) female and 366 (42.5%) male (Table 1). We found no difference in sex distribution between total MAC and *M. abscessus* case-patients of all ages (p = 0.934). Prevalences of MAC, *M. abscessus*, and total MAC/*M. abscessus* cases were higher for female than male case-patients >65 years of age but were similar compared with all other case-patients <65 years (Figure 1). 

**Table 1:** Demographic characteristics of case-patients with MAC and *Mycobacterium abscessus*, by isolate, Virginia, USA, 2021–2023*

Variable	**All**	**MAC isolates**	***M. abscessus* isolates**	**p value†**
Total	864	714	150	
Age, median, y (IQR)	69 (51–87)	69 (52–86)	64 (35–97)	<0.001
Age group, y				
0–18	18 (2.1)	14 (2.0)	4 (2.7)	
18–64	312 (36.1)	240 (33.6)	72 (48.8)	
≥65	534 (61.8)	460 (64.4)	74 (49.3)	
Sex				
F	497 (57.5)	412 (57.7)	85 (56.7)	0.934
M	366 (42.4)	302 (42.3)	65 (42.7)	

*Values are no. (%) except as indicated. MAC, *Mycobacterium avium *complex; IQR, interquartile range †p values given for differences in median age and differences in sex distribution between MAC isolates by Mann-Whitney U and *M. abscessus* by χ^2^ tests.

### Geographic Distribution

**Figure 2:**
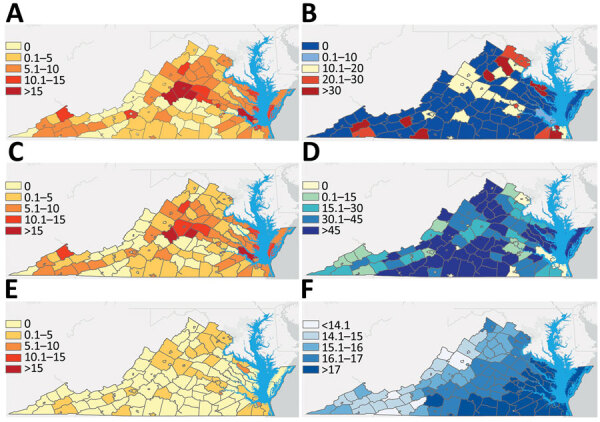
Geographic distribution and variables of interest for Mycobacterium avium *complex (*MAC) and *Mycobacterium abscessus* infections, Virginia, USA, 2021–2023. County-level prevalence (cases/100,000 person-years) of A) MAC/*M. abscessus*; C) MAC; and E) *M. abscessus*. B) *M. abscessus* distribution as a percentage of total MAC/*M. abscessus* infections. D) Percentage of residents using self-supplied water. F) Saturated water vapor pressure in millibars.

Rates of MAC/*M. abscessus* infections varied significantly by locality, driven by differences in distribution of MAC infections (Figure 2). MAC/*M. abscessus* cases clustered throughout the state (Moran *I* = 0.219, p<0.001) similar to MAC (Figure 2 panel C; Moran *I* = 0.210, p<0.001), especially in the central counties of the Piedmont region and on several peninsulas on Chesapeake Bay in the Tidewater region (Figure 2, panels A, C); we found no clear clustering of *M. abscessus* cases (Moran *I* = 0.01, p = 0.663) (Figure 2, panel E). We did find clustering in rates of self-supplied water use (Moran’s *I* = 0.189, p<0.001) and mean annual saturated vapor pressure (Moran *I* = 0.820, p<0.001) (Figure 2, panels D, F). Self-supplied water use appeared to cluster in the more rural south-central parts of the Piedmont region; saturated vapor pressure was highest in the Tidewater region in the southeastern part of the state. 

A regression model of county-level prevalence of MAC/*M. abscessus* infections (Table 2) showed saturated vapor pressure to be associated with prevalence of MAC/*M. abscessus* infections. Each 1 millibar increase in mean annual saturated vapor pressure resulted in a 41.4% increase in expected count of MAC/*M. abscessus* infections (prevalence ratio [PR] 1.414, 95% CI 1.011–1.980; p = 0.043), whereas each 1% increase in the proportion of the county population using self-supplied water resulted in a 69.6% decrease in expected MAC/*M. abscessus* infections (IRR 0.304, 95% CI 0.098–0.950; p = 0.041). Other population-level variables included in the model were not significantly related to MAC/*M. abscessus* prevalence rates. A similar model was constructed to evaluate effects of median age, sex, population density, saturated vapor pressure, temperature, precipitation, and proportion of self-supplied water use on prevalence of MAC or *M. abscessus* infections. Saturated vapor pressure was positively associated and self-supplied water use was negatively associated with MAC infection prevalence, but none of those factors was significantly associated with *M. abscessus* infection prevalence. A model constructed to assess relationships between those factors and prevalence of MAC/*M. abscessus* pulmonary disease identified no significant association. 

**Table 2:** Negative binomial regression model of county-level factors associated with county *Mycobacterium avium* complex and *M. abscessus* case prevalence, Virginia, USA, 2021–2023*

Variable	**PR (95% CI)**	**p value**
Sex		
F	1.068 (0.957–1.192)	0.237
M	Referent	
Median age	1.034 (0.968–1.104)	0.319
Population density	0.893 (0.467–1.708)	0.733
Saturated vapor pressure	1.414 (1.011–1.980)	0.043
Groundwater use	0.304 (0.098–0.950)	0.041
Maximum temperature	0.920 (0.771–1.099)	0.358
Precipitation	0.992 (0.976–1.008)	0.312

*NA, not applicable; PR, prevalence ratio

## Discussion 

We report results of our evaluation of local and statewide rates of MAC/*M. abscessus* infection in Virginia using real-time, laboratory-based monitoring. We found that average annual prevalence of MAC/*M. abscessus* in Virginia over the study period was 6.19 cases of MAC/*M. abscessus* infection per 100,000 population and 2.37 cases of MAC/*M. abscessus* lung disease per 100,000 population. More case-patients were female than male, and most were older persons (median age 69 years), consistent with known demographics associated with NTM infection. Of note, we demonstrated significant geographic clustering of MAC/*M. abscessus*. We found increases in saturated water vapor pressure strongly associated with prevalence and self-supplied water use negatively associated with prevalence at the county level, independent of population density. 

Characterizing the epidemiology of NTM remains challenging, often because of underreporting. Multiple studies have demonstrated the limitations of using diagnostic billing (International Classification of Diseases [ICD]) codes to identify rates of NTM disease. Barriers include lack of clinician familiarity with NTM diagnostic characteristics and variable rates of need for active antimicrobial therapy, which might not be necessary for treatment of NTM lung disease, unlike for many other infectious diseases (*20*,*21*). Several additional recent studies have evaluated laboratory-based surveillance of NTM, including 1 study from a CDC surveillance program (*22*). Our study differed from that study in multiple ways. Of note, we included data from a state in the southeastern United States, a region not represented in the CDC surveillance data, and gathered comprehensive surveillance data for the entire state from statewide laboratories rather than individual sentinel laboratories. Our prevalence estimate for MAC/*M. abscessus* pulmonary disease (2.37/100,000 population) was lower than overall NTM incidence seen in the CDC study (6.1/100,000 population). That difference might be because we included only MAC and *M. abscessus*, not other NTM, or that we included all laboratories statewide rather than only laboratories serving referral centers. Other recent studies based on statewide data from Missouri (*23*) and Wisconsin (*24*) have used laboratory-based surveillance. Comparing prevalence rates based on our data with rates from those other studies was difficult because of differences in methodology and inclusion criteria. The Missouri study (*23*) reported aggregate period rates. The Wisconsin study (*24*) reported an overall average annual NTM incidence of 22.1–22.4 cases/100,000 persons but included repeat positive samples from individual persons as separate cases. In multivariate modeling across those studies, socioeconomic factors were found to be associated with NTM rates in the Wisconsin study but not the Missouri study. We lacked access to those data from patients in our cohort. Our study also differed from the Missouri and Wisconsin studies in that it was set in the southeastern rather than midwestern United States. In addition, we included environmental exposure variables not evaluated in the Missouri and Wisconsin studies (*23**,**24*). 

We found a higher percentage of *M. abscessus* (17.4%) among total MAC/*M. abscessus* infections than other studies of distribution of NTM based on aggregate data (*25*), possibly because we excluded NTM species other than MAC and *M. abscessus*. Still, a recent study showed a range of 4.5%–21.7% widely distributed across the United States for *M. abscessus* (*26*). The southeast had the highest proportion of *M. abscessus* among NTM species of any US region (*26*), but particularly given the clinical severity of *M. abscessus* lung disease, its considerable antimicrobial resistance, and the difficulty of managing antimycobacterial therapy, further research is needed to understand why *M. abscessus* appears to be so prevalent in that region. 

Our study explored associations between MAC/*M. abscessus* infections and local-level environmental exposures. Previous data have shown that variations between locations in temperature, rainfall, flooding, and drought are associated with prevalence of NTM (*27*). Saturated vapor pressure has been shown to be the climate variable most closely associated with NTM prevalence (*6*,*7*). In our study, mean annual saturated vapor pressure was highest in the Tidewater region in the southeastern part of the state and correlated with higher local prevalence of MAC/*M. abscessus*. Of note, saturated vapor pressure is expected to increase globally with ongoing trends in climate change, highlighting the need to understand how those changes might relate to risks of developing NTM lung disease. 

We also examined the relationship between drinking water sources and MAC/*M. abscessus* prevalence. NTM have been more commonly isolated from central water distribution system than groundwater sources, but this comparison has not been tested epidemiologically (*28*). However, several studies have shown piping from central household water sources to be a pathway for NTM infection (*29*,*30*). The source of household water is thought to be critical, with NTM rarely found in samples of clean groundwater (*31*). Here, we found increased use of self-supplied water (mostly well water) to be associated with lower rates of MAC/*M. abscessus* infections in a given locality even after adjusting for population density. Based on our data, the effect size associated with water sources was even larger than with environmental variables, suggesting that water source might constitute a substantial factor in acquiring NTM. 

As with many studies based on laboratory surveillance, our study was limited by a lack of individual-level data regarding water sources and behavioral variables, and we assumed that residential postal codes best reflect the location of a person’s greatest source of exposure to water for drinking and bathing. However, environmental (*31*) and household (*29*,*32*) surveillance data from our study support that water vapor pressure and types of water source might be factors in acquiring NTM. We also considered that the location of referral centers, particularly the cluster of counties surrounding a large academic hospital in central Virginia. might have biased our observation of geographic clustering. However, 1 study of NTM clustering across the United States found that neither physician-to-patient ratio nor referral center proximity within an area was associated with local variations in clustering of NTM prevalence (*33*). In addition to the modest underestimate of NTM lung disease when considering only laboratory-based microbiologic criteria (*16*), MAC and *M. abscessus* represented only 73.6% of pathogenic pulmonary NTM isolates in Virginia based on earlier data from our group (*34*), and thus NTM lung disease likely carries a greater total population burden than we report. Furthermore, given our study design, we could not conclusively establish causation with regards to the association between exposure variables and outcomes of interest. Finally, although recent data were available, we matched covariates only spatially, not temporally. 

In summary, we found a high proportion of NTM isolates in Virginia were MAC. Local clustering of MAC/*M. abscessus* infections within Virginia during the study period might be explained by differences in household water sources and saturated water vapor levels. Future studies of the geographic distribution of NTM should highlight variations in the distribution of different NTM species; additional controlled studies are needed to explore those factors and assess the effects of other individual-level exposures that might be related to developing NTM lung disease. Our findings suggest that a better understanding of geographic clustering and environmental water exposures related to NTM could help inform future monitoring activities and development of prevention and control efforts targeted to populations most at risk. 
